# Human ACE2 receptor polymorphisms and altered susceptibility to SARS-CoV-2

**DOI:** 10.1038/s42003-021-02030-3

**Published:** 2021-04-12

**Authors:** Kushal Suryamohan, Devan Diwanji, Eric W. Stawiski, Ravi Gupta, Shane Miersch, Jiang Liu, Chao Chen, Ying-Ping Jiang, Frederic A. Fellouse, J. Fah Sathirapongsasuti, Patrick K. Albers, Tanneeru Deepak, Reza Saberianfar, Aakrosh Ratan, Gavin Washburn, Monika Mis, Devi Santhosh, Sneha Somasekar, G. H. Hiranjith, Derek Vargas, Sangeetha Mohan, Sameer Phalke, Boney Kuriakose, Aju Antony, Mart Ustav Jr, Stephan C. Schuster, Sachdev Sidhu, Jagath R. Junutula, Natalia Jura, Somasekar Seshagiri

**Affiliations:** 1 Research and Development Department, MedGenome Inc, Foster City, CA USA; 2 Cardiovascular Research Institute, University of California San Francisco, San Francisco, CA USA; 3 Department of Cellular and Molecular Pharmacology, University of California San Francisco, San Francisco, CA USA; 4 MedGenome Labs Ltd., Bangalore, Karnataka India; 5 Department of Molecular Genetics, and the Terrence Donnelly Center for Cellular and Biomolecular Research, University of Toronto, Toronto, ON Canada; 6 ModMab Therapeutics, Foster City, CA USA; 7 ModMab Therapeutics, Accelerator for Donnelly Collaboration, University of Toronto, Toronto, ON Canada; 8 Wellcome Sanger Institute, Cambridge, UK; 9 Center for Public Health Genomics, University of Virginia, Charlottesville, VA USA; 10 Midwestern University, Glendale, AZ USA; 11 Department of Molecular Biology, SciGenom Labs Pvt Ltd, Kochi, Kerala India; 12 AgriGenome Labs Private Ltd, Kochi, Kerala India; 13 Singapore Centre for Environmental Life Sciences Engineering, Nanyang Technological University, Singapore, Singapore; 14 SciGenom Research Foundation, Bangalore, Karnataka India

**Keywords:** Proteins, Molecular biology

## Abstract

COVID-19 is a respiratory illness caused by a novel coronavirus called SARS-CoV-2. The viral spike (S) protein engages the human angiotensin-converting enzyme 2 (ACE2) receptor to invade host cells with ~10–15-fold higher affinity compared to SARS-CoV S-protein, making it highly infectious. Here, we assessed if *ACE2* polymorphisms can alter host susceptibility to SARS-CoV-2 by affecting this interaction. We analyzed over 290,000 samples representing >400 population groups from public genomic datasets and identified multiple ACE2 protein-altering variants. Using reported structural data, we identified natural *ACE2* variants that could potentially affect virus–host interaction and thereby alter host susceptibility. These include variants S19P, I21V, E23K, K26R, T27A, N64K, T92I, Q102P and H378R that were predicted to increase susceptibility, while variants K31R, N33I, H34R, E35K, E37K, D38V, Y50F, N51S, M62V, K68E, F72V, Y83H, G326E, G352V, D355N, Q388L and D509Y were predicted to be protective variants that show decreased binding to S-protein. Using biochemical assays, we confirmed that K31R and E37K had decreased affinity, and K26R and T92I variants showed increased affinity for S-protein when compared to wildtype ACE2. Consistent with this, soluble ACE2 K26R and T92I were more effective in blocking entry of S-protein pseudotyped virus suggesting that ACE2 variants can modulate susceptibility to SARS-CoV-2.

## Abstract (web summary)

Suryamohan, Diwanji, Stawiski et al. identify natural ACE2 variants that are predicted to alter virus–host interactions. They find that soluble ACE2 K26R and T92I variants are more effective in blocking the entry of SARS-CoV-2 S-protein pseudotyped virus, compared to wild-type ACE2. This study suggests that ACE2 variants may modulate the host susceptibility to SARS-CoV-2.

## Introduction

Coronaviruses (CoVs) are widely distributed in nature and pose a serious threat to humans and a range of mammalian hosts, causing respiratory, gastrointestinal, and central nervous system diseases^1^. CoVs are enveloped non-segmented positive-sense single stranded RNA viruses and are classified into α−, β−, γ−, and δ-CoVs^1^. While α- and β-CoVs infect mammals, the γ- and δ-CoVs generally infect birds^1^. Previously, α-CoVs HCoV-229E and HCoV-NL63, and β-CoVs HCoV-HKU1 and HCoV-OC43 have been found to infect humans leading to mild symptoms^1,2^. More recently, three β-CoVs: severe acute respiratory syndrome coronavirus (SARS-CoV) in 2003^1,3^, Middle-East respiratory syndrome coronavirus in 2012 (MERS-CoV)^1,4^, and SARS-CoV-2 in 2019^5–7^ have crossed the species barrier to infect humans resulting in respiratory illnesses including pneumonia that can be fatal.

SARS-CoV-2 is a novel coronavirus (2019-nCoV) first reported in December 2019 and is the cause of an ongoing global pandemic^5–7^. It has infected over 130 million people in 181 countries leading to over 2.8 million deaths as of April, 2021^8^. Sequence analysis of the SARS-CoV-2 genome revealed that it is closer to the bat CoV RaTG13 (96.2% identical) than to SARS-CoV (79.5% identical) that was responsible for the 2003 epidemic, suggesting that this novel virus originated in bats independently before jumping to humans either directly or through a yet to be determined intermediary host^9^.

As with SARS-CoV and a related α-coronavirus NL63 (HCoV-NL63), SARS-CoV-2 employs the human ACE2 cell surface protein as a receptor to gain entry into cells^10–15^. The virus surface spike glycoprotein (S-protein) constitutes a key determinant of viral host range and contains two domains, S1 and S2, which are separated by a protease cleavage site^1^. A successful host cell invasion by the virus involves direct binding of the virus S1 receptor-binding domain (RBD) to the host ACE2 peptidase extracellular domain (PD), exposing the S1-S2 inter-domain protease site that upon cleavage by host proteases, leads to S2-mediated virus-host cell membrane fusion^1,12,16–18^.

The SARS-CoV-2 S-protein is 98% identical to the bat CoV RaTG13 S-protein, with the exception of an insertion that is also absent in the SARS-CoV S-protein in the S1/S2 inter-domain protease cleavage site. This difference has been proposed to alter SARS-CoV-2 tropism and enhance its transmissibility^19^.

Several structural studies involving the SARS-CoV-2 S-protein RBD and ACE2 PD have identified key residues involved in their interaction^19–22^. The S-protein RBD was reported to bind ACE2 PD with ~10- to 20-fold higher affinity (~15 nM) when compared to the SARS-CoV S-protein RBD^20,22^, potentially contributing to the high rate of SARS-CoV-2 infection.

As the interactions between the ACE2 receptor and S-protein RBD interface are critical for the cellular entry of the virus, we wanted to ascertain if there were natural ACE2 variations that would decrease or increase its affinity to the S-protein RBD and may thus protect or render individuals more susceptible to the virus. Consistent with this possibility, a saturation mutagenesis screen of select ACE2 PD residues identified variants that showed enhanced or decreased binding to S-protein^23^.

In this study, we have analyzed ACE2 protein-altering variants in a large cohort of human population groups and identified polymorphisms that either likely protect or render individuals more susceptible to the virus. Understanding these changes at the molecular level, combined with the genotype and epidemiological data will allow the elucidation of population risk profiles and also help advance therapeutics such as a rationally designed soluble ACE2 decoy-receptor for treatment of COVID-19.

## Results

### Human ACE2 population polymorphism

**Fig. 1:**
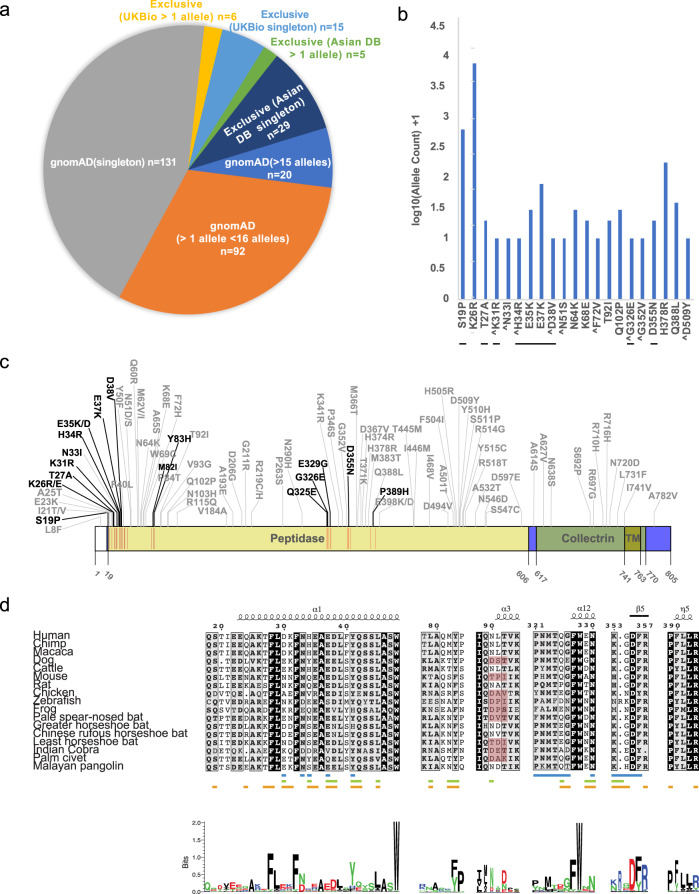
ACE2 polymorphisms. **a** Pie chart representing protein altering variations in ACE2 by allele count and source. **b** Log base 10 pseudo count adjusted (+1) observed ACE2 allele counts of mutants predicted to impact S-protein binding. Singletons are marked with a ^ and direct S-protein contact residues are underlined. **c** ACE2 protein domain showing positions with polymorphisms that can alter SARS-CoV-2 S-protein binding. Recurrent polymorphisms (*n* > 1) that were predicted to not impact S-protein binding are shown in light grey. Residues within the ACE2 peptidase domain (PD) known to interact with viral S-protein are shown as red vertical lines within the peptidase domain in the ACE2 diagram. **d** Multiple sequence alignment of the S-protein interacting ACE2 sequence from indicated species. ACE2 NxT/S glycosylation motif disrupted in dog, rat, palm civet, and several bat ACE2 is highlighted in red. ACE2 residues that mediate contact with NL63-CoV, SARS-CoV and SARS-CoV-2 are shown as blue, green and orange bars, respectively.

The SARS-CoV-2 S-protein interacts with the ACE2 PD to enter human host cells. Analysis of the RBD domain of SARS-CoV-2, SARS-CoV and bat CoV RaTG13 S-proteins identified changes that have increased the affinity of CoV-2 S1 RBD to human ACE2, which likely contributes to its increased infectivity^20,22^. It is very likely that there exists ACE2 variants in human populations, though not under selection, that may increase or decrease its affinity to SARS-CoV-2 S-protein and thereby render individuals more resistant or susceptible to the virus. To investigate this, we assessed *ACE2* protein-altering variations from a number of databases including gnomAD^24^, RotterdamStudy^25^, ALSPAC^26^ and Asian-specific databases, namely GenomeAsia100k^27^, TOMMO-3.5kjpnv2^28^, IndiGen (https://indigen.igib.in/), and HGDP^29^ (Supplementary Data 1). We found a total of 298 unique protein altering variants across 256 codons distributed throughout the 805 amino acid long human ACE2 (Fig. 1a, b and Supplementary Fig. 1, Supplementary Data 1). The most frequent variant, N720D (1.6% allele frequency; *n* = 3054, gnomAD), was found in the C-terminal collectrin domain that is not involved in the SARS-CoV-2 S-protein interaction. Overall, we found human *ACE2* receptor polymorphisms to be low with a weighted mean Fst (fixation index) value of 0.0168. and the *ACE2* PD showed even more reduced variation (Wilcoxon *p*-value = 0.0656, *n* = 65, Supplementary Fig. 2, see “Methods” section).

Further, genealogical estimation of variant age (GEVA) suggests that *ACE2* coding variants are more recent (Supplementary Fig. 3). Although *ACE2* has been reported to be highly intolerant of loss-of-function variants (pLI = 0.9977, gnomAD; Supplementary Fig. 2, see “Methods” section)^24^, we observed 5 predicted LOF singleton alleles (Supplementary Data 1).

**Fig. 2:**
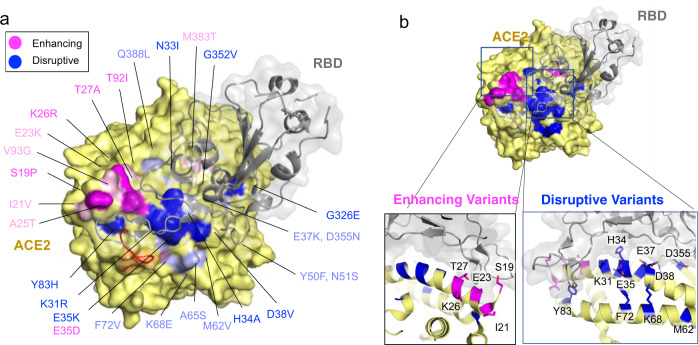
Human ACE2 polymorphisms mapped to the structure of human ACE2 in complex with the SARS-CoV-2 RBD. Residues in ACE2 showing polymorphic variation in human populations were mapped on to the structure of the ACE2/SARS-CoV-2 RBD (PDB: 6VW1) and colored according to their effect on the predicted affinity to SARS-CoV-2 RBD. Polymorphisms that were predicted to enhance the binding between ACE2 and the S-protein are colored in magenta. Polymorphisms that are predicted to disrupt the binding between ACE2 and the S-protein are colored in dark blue. The variable loop in the ridge binding motif consisting of residues V483 and E484 is shown in red. This region in the structure (PDB: 6LZG) is zoomed-in to show variants predicted to enhance or disrupt the ACE2 – SARS-CoV-2 interaction.

Structural studies involving SARS-CoV and SARS-CoV-2 S-protein in complex with human ACE2 have identified three regions in a ~120 amino acid claw-like exposed outer surface of human ACE2 (ACE2-claw) that contributes to its binding to the S-protein^19–22^. The key residues at the ACE2 S-protein-RBD interface include S19, Q24, T27, F28, D30, K31, H34, E35, E37, D38, Y41, Q42, L45, L79, M82, Y83, T324, Q325, G326, E329, N330, K353, G354, D355, R357, P389, and R393 (Fig. 1b). Mutagenesis of four residues, namely M82, Y83, P84 and K353, in the S-protein-binding interface of rat ACE2 was sufficient to convert rat ACE2 into a human SARS-CoV receptor, further indicating the importance of this region in determining the host range and specificity of CoVs^30^. Considering these findings, we focused on variants within the human ACE2-claw S-protein RBD-binding interface and identified protein alterations in 44 codons that resulted in 49 unique variants for a total of 968 allelic variants. This included K26R, the second most frequent human ACE2 protein-altering variant (0.4% allele frequency; allele count = 797, gnomAD), S19P, T27A, K31R, N33I, H34R, E35K, E37K, D38V, N51S, N64K, K68E, F72V, T92I, Q102P, G326E, G352V, D355N, H378R, Q388L, and D509Y (Figs. 1c, d, 2 and Supplementary Data 1). These variants could potentially increase or decrease the binding affinity of ACE2 to the S-protein and thereby alter the ability of the virus to infect the host cell.

### Structural evaluation of ACE2 polymorphism

To investigate the effect of the ACE2 polymorphisms on receptor recognition by the SARS-CoV-2 RBD, we modeled the identified ACE2 variants using published cryo-EM and crystal structures of ACE2/SARS-CoV-2 RBD complexes^19–22^. Based on the evaluation of the structures and a functional analysis of a synthetic human ACE2 mutant library for RBD-binding affinity^23^, we broadly classified ACE2 polymorphic variants into two categories with respect to their predicted effect on ACE2-RBD-binding as enhancing or disrupting (Fig. 2 and Supplementary Data 2, 3). These two groups of polymorphic variants mapped onto the ACE2 structure remarkably segregate into two distinct clusters at the ACE2/CoV-2 RBD interface (Fig. 2a). The predicted enhancing variants cluster to the ACE2 surface most proximal to the receptor-binding ridge of CoV-2 RBD (Fig. 2b) whereas the majority of the predicted disrupting variants reside centrally on the two major ACE2 α-helices that substantially contribute to the buried surface area at the interface (Fig. 2b).

The spatial segregation of the functionally different ACE2 variants can be structurally explained. The loop conformation in the receptor-binding ridge of SARS-CoV-2 differs from that of SARS-CoV owing to the presence of bulky residues (V483 and E484) in the loop^22^. This feature allows the SARS-CoV-2 loop to extend further towards ACE2 establishing more extensive contacts with the receptor (Fig. 2a). Hence, natural ACE2 variants in this region could be exploited by the CoV-2 loop, increasing susceptibility to viral infection. In contrast, most interactions that CoV-2 makes with the core of the ACE2 interface are centered on two α-helices (α1 and α2) and are not unique to CoV-2. They encompass what seem to be critical binding hotspots, discussed below, and thus centrally located polymorphic variants are more likely to reduce viral recognition.

**Fig. 3:**
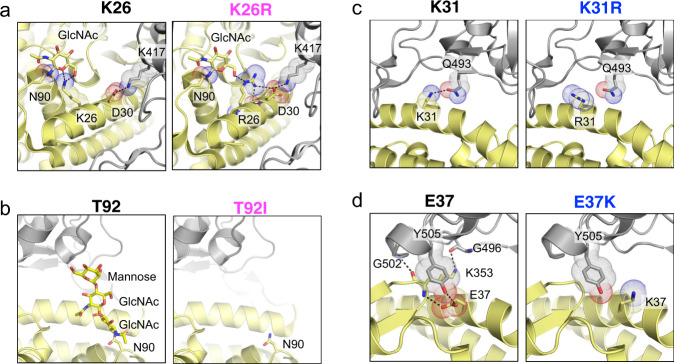
Structural basis of interaction between human ACE2 polymorphic variants and SARS-CoV-2 S-protein. **a** K26R, **b** T92I, **c** K31R and **d** E37K. Interaction mapping done using PDB structure 6VW1.

By far the most frequent variant identified in our data, K26R (~0.4% allele frequency), is predicted to enhance ACE2 affinity for SARS-CoV-2. Based on its structure, K26 establishes polar contacts with the first mannose moiety of the partially resolved ACE2 N90-linked glycan and likely stabilizes the position of the glycan relative to the native protein (Fig. 3a). The N90-linked glycan emerges as an important determinant of CoV-2 infectivity and may diminish ACE2 affinity for the RBD possibly through steric hindrance imposed by branching of the sugar modifications^31^. We predict that K26R would abrogate stabilizing polar contacts with N90, impairing coordination of the glycan (Fig. 3a) and lead to an increase in the affinity of the virus to the ACE2 receptor. Further, R26 is now primed to establish backbone and side chain interactions with ACE2 D30 which then is poised to build a salt-bridge with CoV-2 RBD K417 (Fig. 3a). In this scenario, the net effect of K26R polymorphism is the stabilization of core α-helices that increases ACE2 binding affinity to CoV-2 RBD at the cost of glycan rigidity. Another variant likely to enhance affinity through a similar mechanism is T92I. T92I directly targets the glycosylation consensus sequence (_90_NXT_92_). This mutation would eliminate post-translational modification by the N90-linked glycan providing an opportunity for a more optimal ACE2/RBD interaction (Fig. 3b). The predicted effect of the T27A variant is increased hydrophobicity at the interface which could contribute to an increase in binding affinity. Similar destabilizing patterns are predicted for the variants S19P and E23K (Supplementary Data 3).

The vast majority of predicted disruptive ACE2 polymorphic variants map to the core α-helical bundle of ACE2 and to residues known to form contacts with the RBD. There are two key hotspot residues in the α-helical bundle of the ACE2 interface that are important for CoV-2 RBD-binding: K31 and K353. To enable interaction with the virus, these charged residues need to be accommodated in a largely hydrophobic environment at the binding interface and hence their neutralization is critical to the binding of coronavirus RBDs to human ACE2^22,32,33^. A recent elegant study^22^ showed that SARS-CoV-2 S-protein is more effective in neutralization of the lysine hotspots than SARS-CoV due to the presence of Q493 and L455 that stabilize K31, and N501 that stabilizes K353. Interestingly, K31R is one of the human ACE2 polymorphisms that we identified. Introduction of an arginine not only maintains the positive charge at position 31 but is also predicted to break an interaction with Q493 in the RBD (Fig. 3c and Supplementary Fig. 4a) and destabilize the charge-neutralizing interaction with the virus. Similarly, E37, also located in the core α-helical bundle coordinates polar contacts with RBD Y505, RBD G502, and ACE2 K353 (Fig. 3d). The E37K variant we identified is predicted to disrupt these critical interactions by removing the polar intramolecular interaction with ACE2 K353 and stabilizing contacts with the RBD (Fig. 3d). Thus, individuals carrying K31R or E37K ACE2 variants are predicted to be less susceptible to SARS-CoV-2 infection. While we did not identify any polymorphic variants at residue K353, we detected an ACE2 mutation at D38 (Supplementary Data 1), which forms an electrostatic interaction with K353 (Supplementary Data 3). This mutation, D38V, would compromise the neutralizing effect of the K353-D38 interaction at the interface and is predicted to reduce binding affinity between the virus and the host receptor.

Another recurrent polymorphism in ACE2 maps to residue E35 where the glutamate is replaced by a lysine (E35K; Supplementary Data 2). E35 establishes a critical polar contact with SARS-CoV-2 S-protein residue Q493, which is predicted to be attenuated in the presence of the positively charged lysine (Supplementary Fig. 4b). Interestingly, the ACE2 E35 interaction is not conserved between SARS-CoV and SARS-CoV-2 S-proteins^22^ and hence we predict that E35K could offer selective protection from the SARS-CoV-2 infection in individuals carrying this variant in a manner akin to H34R (Supplementary Fig. 4c) which similarly also results in a loss of interface polar contacts. Another interesting polymorphism at position 83 results in a Y83H alteration. Notably, in mouse, residue 83 is phenylalanine (Fig. 1d)^34^. Residue F/Y83 underlies a hydrophobic pocket into which F486 from SARS-CoV-2 RBD is inserted (Supplementary Fig. 4d). This is another unique interaction involving ACE2 and the SARS-CoV-2 RBD F486 that is absent in SARS-CoV RBD where the equivalent residue is a leucine^22^. The polymorphism that replaces Y83 with a polar histidine will compromise the hydrophobic character of this unique pocket in addition to removing a polar contact with N487 (Supplementary Fig. 4d), potentially offering selective protection from SARS-CoV-2 infection.

### Altered affinity of ACE2 variants for SARS-CoV-2 S-protein

To validate our structural predictions, we measured the effect of select ACE2 polymorphisms on its binding affinity to CoV-2 S-protein. We expressed and purified the S1 subunit of the S-protein, CoV-2 S-RBD, and a trimer stabilized form of S-protein (S-trimer; Supplementary Fig. 5). We also recombinantly produced His-tagged monomeric and Fc-tagged dimeric forms of the extracellular domain of wildtype ACE2 (WT) and variant forms of ACE2 (S19P, K26R, K31R, E37K and T92I; Supplementary Fig. 5). These variants were selected based on their population frequency and the predicted effect on their interaction with S-protein.

**Fig. 4:**
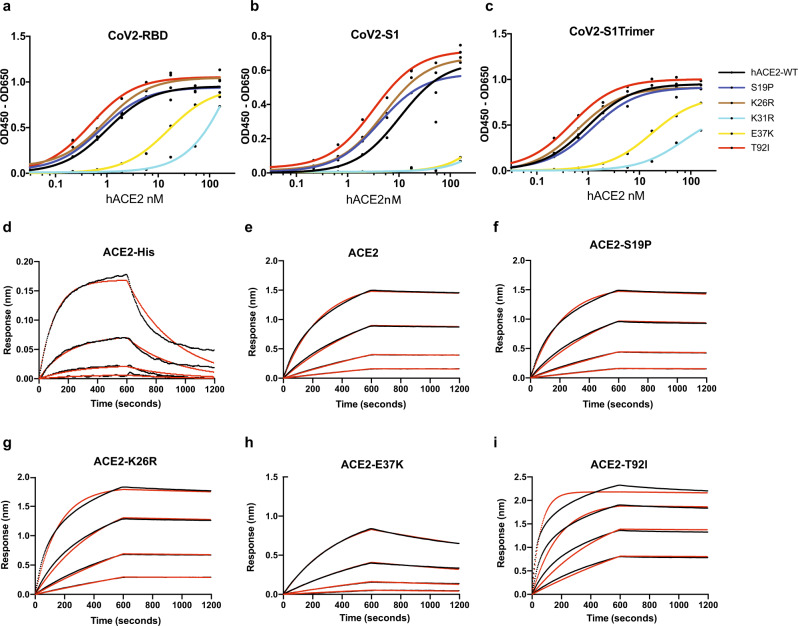
Binding affinity of SARS-CoV-2 S-RBD, S1 and S-trimer. **a**–**c** ELISA assay measuring the affinity of indicated ACE2 WT or variants for SARS-CoV-2 S-RBD (**a**), S1 (**b**) and S-trimer (**c**). **d**–**i** Sensorgrams for the binding of a 3-fold dilution series of monomeric and dimeric ACE2 variants to streptavidin-captured SARS-CoV-2 RBD are provided (black). Fits to the empirical binding curves are shown overlaid (red).

We tested the affinity of these ACE2 variants against a panel of S-protein constructs using enzyme-linked immunosorbent assay (ELISA) and/or bio-layer interferometry (BLI; Fig. 4 and Table 1). For ELISAs, we used dimeric ACE2-Fc to assess its binding to the S-protein variants. We found the ACE2-Fc WT dimer bound to the isolated S-RBD (EC50 1.01 nM) and S-trimer (EC50 0.95 nM) more strongly compared to the S1 subunit (EC50 10.4 nM) (Fig. 4a–c and Table 1). This is consistent with previous studies that showed a decreased ACE2 affinity for SARS-CoV S1 subunit compared to S-RBD, indicating a conformational difference between these variants^12,16,35^. In the trimeric state, in contrast to the monomeric full-length S1-protein, the RBD within the S1 subunit in one or more of the constituent S-proteins is known to adopt a receptor-accessible “RBD-out” conformation, supporting its high affinity for ACE2 that is comparable to that observed for isolated RBD^20,21,36^.

**Table 1:** SARS-CoV-2 S-protein affinity for ACE2 variants.

	ELISA			BLI						PVNA
hACE2 variant	EC50, nM [ACE2-Fc]			*K*_d_ (nM) [S-RBD]	*K*_on_ (10^5^ M^-1^ s^−1^)	*K*_off_ (10^5^ s^−1^)	*K*_d_ (nM) [S-RBD]	*K*_on_ (10^5^ M^−1^ s^−1^)	*K*_off_ (10^5^ s^−1^)	*K*_i_ (nM)
	S-RBD	S1	S-trimer	RBD vs ACE2-Fc			RBD vs ACE2-His			ACE2-Fc
WT	1.01 ± 0.04	10.4 ± 0.05	0.95 ± 0.03	1.21 ± 0.2	0.31 ± 0.1	3.74 ± 0.6	117 ± 11	0.26 ± 0.1	304 ± 7	>1000
S19P	0.77 ± 0.08	4.16 ± 0.13	1.20 ± 0.03	1.05 ± 0.1	0.55 ± 0.1	5.79 ± 0.3	80 ± 3	0.46 ± 0.1	370 ± 4	92 ± 30
K26R	0.89 ± 0.11	5.04 ± 0.02	0.62 ± 0.06	0.36 ± 0.1	0.76 ± 0.1	2.80 ± 0.5	114 ± 8	0.27 ± 0.1	309 ± 5	30 ± 13
K31R	298 ± 0.64	NB	73 ± 0.07	ND	ND	ND	ND	ND	ND	ND
E37K	15.8 ± 0.03	NB	17.6 ± 0.02	22.7 ± 0.4	0.18 ± 0.1	41.0 ± 0.4	NB	NB	NB	NB
T92I	0.48 ± 0.03	3.22 ± 0.03	0.47 ± 0.04	0.13 ± 0.1	2.80 ± 0.1	3.7 ± 0.8	87 ± 6	0.37 ± 0.1	321 ± 5	53 ± 14

*BLI* bio-layer interferometry, *PVNA* Pseudovirus neutralization assay, *NB* no binding, *ND* not determined (for K31R, BLI values could not be determined given the low affinity observed in ELISA).

Using BLI, we also tested the interaction between monomeric ACE2 WT or dimeric ACE2-Fc WT and S-RBD and found that ACE2-Fc (*K*_d_ = 1.21 nM) bound more tightly to S-RBD compared to monomeric ACE2 (*K*_d_ = 117 nM) (Table 1). Recent structural studies show ACE2 interacts with the S-protein as a dimer and hence the observed higher affinity of the ACE2 dimer is likely critical for a productive infection^21,37^.

Next, using ELISA and/or BLI assays, we measured the affinity of the S-RBD or S-trimer for ACE2-Fc variants (Fig. 4 and Table 1). While the S19P ACE2-Fc mutant had marginally increased affinity compared to WT ACE2-Fc (*K*_d_ 1.21 vs 1.05 nM), ACE2 K26R mutant showed higher affinity for S-RBD (*K*_d_ = 0.36 nM) and S-Trimer (EC50 0.62 nM) (Table 1). The 4- to 5-fold increased affinity of K26R mutation for S-RBD relative to ACE2 WT was in part due to a >2-fold enhancement in its on-rate (*K*_on_ 0.76 vs 0.31 × 10^5^ M^−1^ s^−1^) combined with a ∼25% reduction in its off-rate (Table 1). Similarly, the T92I, glycosylation site mutant of ACE2-Fc, showed an increased affinity for S-RBD (*K*_d_ = 0.13 nM) and S-trimer (EC50 0.47 nM). The pronounced ∼9-fold increase in affinity of ACE2 T92I versus ACE2 WT (*K*_d_ 0.13 vs 1.21 nM) is consistent with a ~9-fold increase in its on-rate (*K*_on_ 2.8 vs 0.31 × 10^5^ M^−1^ s^−1^). In contrast, E37K mutation led to a loss of ACE2 affinity for S-RBD (*K*_d_ = 22.7 nM) and S-trimer (EC50 = 17.6 nM). The ~18-fold decrease in affinity of E37K compared the WT ACE2 is in part due to a ~10-fold increase in its off-rate (*K*_off_ 41 vs 3.74 × 10^−5^ s^−1^). As, observed with E37K, K31R ACE2-Fc had a decreased affinity for S-RBD (EC50 = 298 nM) and S-trimer (EC50 = 73 nM) when compared to WT ACE2-Fc.

### Affinity-enhancing ACE2 variants are more effective in virus neutralization

**Fig. 5:**
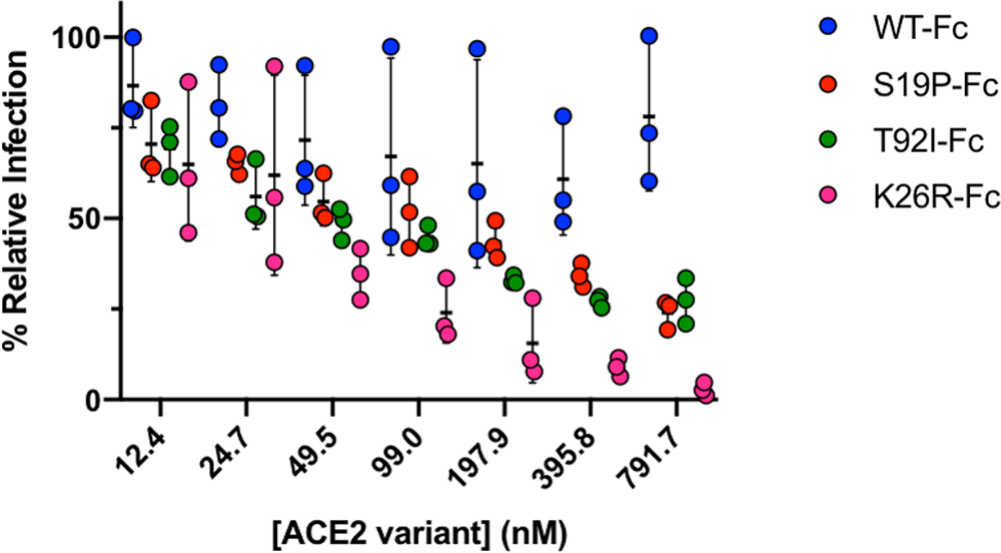
ACE2 variants block pseudotyped SARS-CoV-2 virus infection. Inhibition of pseudovirus entry into HEK293T cells by ACE2 WT and mutants S19P, T92I, and K26R. A range of seven different concentrations of Fc-tagged ACE2 proteins were mixed with pseudoviruses and the infectivity was measured by the luciferase signal as an indication of the amount of virus entering the cells. Data are presented as the mean ± SD (standard deviation). Sample size *n* = 3.

We tested the ability of Fc-tagged ACE2 WT, and its variant forms, for their ability to block S-protein pseudotyped virus from entering infection-competent cells (Fig. 5 and Table 1). Among the variants tested, ACE2 K26R, consistent with its increased affinity for S-RBD observed in the biochemical assay, was the most effective (*K*_i_ 30 nM; ~33 fold greater than WT) in blocking viral entry (Fig. 5 and Table 1). Similarly, ACE2 T92I variant was ∼20-fold more potent in blocking the pseudovirus compared to ACE2 WT (*K*_i_ 53 vs >1000 nM; Fig. 5 and Table 1). Interestingly, though ACE2 S19P showed only a minor increase in affinity for S-RBD in the biochemical assay, it was ~11 fold more effective (*K*_i_ > 92 nM) in blocking viral entry, suggesting that a subtle increase in affinity might be sufficient to alter susceptibility.

## Discussion

The host-virus evolutionary arms race over time leads to natural selection that alters the host and the viral proteins allowing both to increase their fitness^38^. In this context, multiple studies have analyzed and identified the origin, evolution and successful adaption of the SARS coronaviruses as human pathogens^9,39^. Viral genome sequencing and analysis has identified bats to be the most likely natural host of origin for both SARS-CoV and the recent SARS-CoV-2^9^. In particular, several studies have focused on the viral S-protein RBD that interacts with its host ACE2 receptor and identified key changes between the bat CoVs and other suspected intermediary host CoVs found in the civet and pangolin^19–22,39,40^. These studies have identified S-protein changes that have rendered the human cells permissive to the SARS-CoV and SARS-CoV-2 infection^19–22,40^.

Thus far, the role of variations in human ACE2 receptor in susceptibility to both SARS CoVs had not been comprehensively examined. While a recent in silico study analyzed limited ACE2 population variation data set and concluded that these polymorphisms did not confer resistance to the virus^34^, other studies have implicated ACE2 variants in altering binding to S-protein^41–45^. In this study, we comprehensively examined human ACE2 variation data compiled from multiple data sets and identified polymorphisms that will either likely render individuals more susceptible to the SARS-CoV-2 or protect them from the virus. Using published protein structures and data from a high-throughput functional mutagenesis screen that used deep sequencing to assess enrichment or depletion of S-protein binding to ACE2 variants (Supplementary Fig. 6), we performed structural modeling to classify ACE2 variants identified in this study based on their effects on susceptibility to SARS-CoV^19–23^.

We identified several ACE2 polymorphic variants that increase ACE2/S-protein interaction including S19P, I21V, E23K, K26R, K26E, T27A, N64K, T92I, Q102P, M383T and H378R (Supplementary Data 1, 2 and 3). Among these, the T92I polymorphism is part of a NxT/S consensus N-glycosylation motif^46^. The ACE2 NxT/S motif, while conserved in 96 out of 296 jawed vertebrates, it is absent or altered in several species, including the civet cat (*Paguma larvata*). The NxT/S motif is altered in several bat species and this includes substitution at N90, presence of a proline at position 91 or any amino acid except serine at T92, any of which will abolish the glycosylation at N90 (Fig. 1d and Supplementary Fig. 7, Supplementary Data 4)^30,31,46,47^. These ACE2 variations are expected to abolish glycosylation at N90^46^. Another mutation that altered the NxT/S motif in human ACE2 to a civet ACE2-like sequence (90-NLTV-93 to DAKI), also expected to abolish the N-glycosylation, was shown to increase the SARS-CoV infectivity and S-protein binding (Fig. 1d)^30^. A previous study showed that the ACE2 N90 renders human cells resistant to civet CoV^30^.

Our structural investigation suggests that the T92I mutation favors improved Cov-2 S-protein binding. Using recombinant T92I mutant ACE2 protein, we showed that it had an increased affinity for S-RBD and also found it to be more effective in blocking virus entry compared to ACE2 WT (Fig. 5 and Table 1). Further, the T92I mutant showed a strong enrichment in a sequencing-based screen for S-protein binders^23^. Thus, the T92I mutation likely renders individuals harboring this mutation more susceptible to the virus. Taken together, these observations suggest that N90 glycosylation site is critical and it could confer protection through glycan shielding. ACE2 N90 glycosylation could also determine the strength and specificity of infection by different CoV viruses.

We also show that another ACE2 residue, K26, plays an important role in controlling the susceptibility to viral infections via a similar mechanism. Our structural analysis has suggested that K26R mutation will weaken coordination of the N90-linked glycan presumably interfering with its ability to shield the host from the viral infection. Our biochemical binding assays confirmed the predicted increased affinity for K26R ACE2 for S-protein. In fact, K26R ACE2 was the most effective among mutants tested for their ability to enhance viral entry, suggesting that this frequent polymorphism, very likely increases susceptibility to SARS-CoV-2 (Fig. 5 and Table 1).

We also found ACE2 variants predicted to reduce ACE2 S-protein interactions and thereby decrease S/ACE2 binding affinity. Variants predicted to reduce ACE2 S-protein interactions and thereby decrease S/ACE2 binding affinity include K31R, N33I, H34R, E35K, E37K, D38V, Y50F, N51S, K68E, F72V, Y83H, G326E, G352V, D355N and Q388L (Supplementary Data 1 and 2). Biochemical binding assays confirmed the decreased affinity of two variants that we tested, K31R and E37K, indicating that these likely are protective polymorphisms.

Overall, we find the *ACE2* population variants, that either increase or decrease susceptibility, to be rare, which is consistent with the overall low number of *ACE2* receptor population level polymorphisms (mean Fst 0.0167). Also, we did not observe any statistically significant difference in *ACE2* variant allele frequency among population groups. The variant alleles also did not show discernable gender distribution differences, even though *ACE2* is a X-linked gene. The SARS-CoV infections and its deadly effects in humans are more recent and thus the pathogenic and protective variants have not been subject to purifying selection and therefore are predictably rare.

The expression levels of ACE2 and its variants in appropriate host tissue may modulate the deleterious effect of the virus. To further understand the importance of the ACE2 variants in susceptibility, it will be important to correlate clinical outcomes with *ACE2* genotypes at population scale. ACE2 K26R, predicted to increase susceptibility to SARS-CoV-2, is found in 8 women and 6 men in the UK Biobank exome sequencing data set. Two of the 6 men tested positive for SARS-CoV-2 infection, representing a (non-significant) 2.4-fold increased odds of infection compared to those who do not carry the variants (Fisher’s exact *p*-value = 0.279). No other variants with predicted binding affinity were found in the UK Biobank participants with both exome sequencing data and COVID-19 test results.

Genetic variation in *ACE2* alone is unlikely to explain the vast variability in infection susceptibility and severity of COVID-19. While a handful of large genome-wide association studies (GWAS) of SARS-CoV-2 infection status have identified additional genetic risk factors^48,49^, the *ACE2* locus shows only weak association in these studies, possibly due to the lack of common variation in the locus. The extremes in COVID-19 clinical symptoms reported range from asymptomatic infected adult individuals to those that show acute respiratory syndrome leading to death^50–52^. This suggests a role for additional factors, including the role of innate and adaptive immunity, besides variation in *ACE2* in modifying disease outcomes.

Currently, there are no approved targeted therapeutics for curing SARS-CoV-2 infection. Therefore, development of therapeutics to treat patients and mitigate the COVID-19 pandemic is urgently needed^52,53^. Several small molecules and neutralizing antibodies for treatment are in development^54,55^. Soluble ACE2 and ACE2-Fc fusion protein have been proposed as decoy SARS-CoV-2 receptor therapeutic^56–58^. Soluble ACE2, as a therapy for pulmonary arterial hypertension, has been shown to be safe in early human clinical studies^59,60^. A rationally designed, catalytically inactive, human ACE2 that carries one or more of the natural variants predicted to show improved binding to SARS viral S-protein RBD could be safely developed as a soluble protein with or without an Fc domain for treatment of COVID-19. Even though a human recombinant soluble ACE2 is in clinical trials to treat SARS-CoV-2 infection^61^, a catalytically inactive soluble ACE2 might be preferred from a safety perspective, as S-protein binding enhances ACE2’s carboxypeptidase activity^62^. Additionally, as ACE2 enzymatic activity modulates multiple biological pathways^63^, a catalytically inactive form should be considered for treating SARS-CoV-2 infection. Such a recombinant ACE2 protein can be engineered to create a pan-CoV neutralizing drug that is broad and can neutralize CoVs that may emerge during future epidemics.

Understanding the natural *ACE2* polymorphism spectrum not only provides information on the SARS-CoV-2 susceptibility but can also be used to generate high affinity, rationally designed soluble ACE2 receptor molecules. Such agents that carry naturally occurring polymorphism(s) will lead to no or low immunogenicity in a drug setting and can be used as a decoy-receptor for treating patients.

## Methods

### Identification of ACE2 polymorphisms

We queried multiple genomic databases including gnomAD^24^ (https://gnomad.broadinstitute.org/), DicoverEHR^64^, RotterdamStudy^25^, ALSPAC^26^ and Asian-specific databases which included GenomeAsia100k^27^, HGDP^29^, TOMMO-3.5kjpnv2^28^ IndiGen (https://indigen.igib.in/) and Other aggregated data for ACE2 protein altering variations in populations groups across the world. The *ACE2* genotypes in this study were from over 290,000 samples representing over 400 population groups across the world.

### Fst analysis

To assess genetic variation in the coding region of *ACE2*, we calculated the fixation index (Fst) from 2381 unrelated individuals across 26 populations in the 1000 Genomes Project Phase 3 and 57,783 female individuals across eight populations in gnomAD. For 1000 Genome data, we used the Weir and Cockerham (1984) method as implemented in vcftools (Version 0.1.17); the weighted Fst were calculated from 88 variants. For gnomAD (v2.1.1), because we only have access to the allele counts, we used the original formulation by Wright (1969)^65^ and reported the weighted mean Fst as described in Bhatia et al. (2013)^66^; 277 variants were used. Because Fst values vary based on variants used (Bhatia et al. 2013^66^), we calculated the Fst in a set of randomly selected genes on the same chromosomes matched by the length decile to use for comparison. To assess if variants in the peptidase domain has lower genetic variation, we used the one-sided Wilcoxon rank-sum test to compare 15 variants in the peptidase domain against 50 variants outside. Variants with Fst < 1e−4 were removed as they were uninformative.

### Genealogical estimation of variant age (GEVA)

We used data from the 1000 Genomes Project^67^ to estimate the time of mutation of all variants located within a 1 Mb region around the *ACE2* gene on Chromosome X, from the female-only subset of 1271 individuals (Supplementary Fig. 3a). As previously described^68^, we performed the analysis using an effective population size of Ne = 10,000, mutation rate *µ* = 1.2 × 10^−8^, and with variable recombination rates according to HapMap2^69^. We used the most recent version of GEVA software (https://github.com/pkalbers/geva/tree/ancallele), which allowed us to provide external information about predicted ancestral and derived allelic states from Ensembl (release 95) to correct model assumptions for all variants on Chromosome X. Variant age is estimated through pairwise analyses between haplotype sequences which may or may not carry the derived allele at a given variant. We analyzed each variant using a maximum of 5000 concordant pairs (carrier and carrier haplotypes) and 5000 discordant pairs (carrier and non-carrier) to achieve high confidence. We further distinguished variants into non-coding, synonymous, and missense variants using the Ensembl Variant Effect predictor (release 95)^70^ and separated variants affecting *ACE2* (*n* = 385) from those outside the *ACE2* gene region (*n* = 9095). The proportion of rare variants (≤0.1% frequency of the derived allele within the sample) was similar in both groups; 19% and 22%, respectively (Supplementary Fig. 3b). For variants outside the *ACE2* gene, we found that 54% of non-coding variants were estimated to have arisen within the last 1000 generations, compared to 75% of synonymous and 80% of missense variants (Supplementary Fig. 3c). This suggests that past selective pressure may have acted more strongly to prune mutations that occur within the coding region of the genome. We found that this signal was more pronounced for missense mutations affecting *ACE2*, where we found 58% of non-coding and 60% of synonymous variants to be younger than 1000 generations, whereas all missense variants were younger than approximately 800 generations. The average age (±SE) of missense variants affecting *ACE2* was 472 (±58) generations, compared to 3016 (±2198) generations for variants outside the *ACE2* gene region. However, the low number of coding variants found within the focal 1 MB region for which we were able to estimate the age (*n* = 43 missense and *n* = 37 synonymous variants) makes such comparisons difficult.

### ACE2 ortholog sequence analysis

A total of 295 Human *ACE2* orthologs were obtained from NCBI (Supplementary Data 4 for accession numbers). A snake ACE2 ortholog protein was obtained from the published Indian cobra genome^71^. Multiple sequence alignment of residues surrounding the ACE2 NxT/S motif was performed using MCoffee (www.tcoffee.org). Phylogenetic trees were constructed using the PhyML webserver (www.phylogeny.fr).

### Structural analysis

Each identified variant was mapped, modeled, and analyzed in Pymol using the recently deposited crystal structures 6VW1 and 6LZG of human ACE2 bound to either chimeric SARS-CoV-2 RBD (6VW1) or complete SARS-CoV-2 RBD (6LZG).

### Cloning and protein expression

Extracellular domain (amino acids 1–615; NP_001358344) of human *ACE2* (hACE2) WT or variants with a c-terminal 8x-His or human-Fc tag was synthesized (IDT, USA) and cloned into a CMV promoter-driven mammalian expression vector. Human codon optimized CoV-2-S-RBD (amino acids 319-541; YP_009724390) sequence with a c-terminal 8x His-tag were synthesized and cloned into a CMV promoter-driven mammalian expression vector. The prefusion SARS-CoV-2 S-protein trimer stabilized ectodomain (amino acids 1–1208; YP_009724390), as previously described^20^, containing K986P, V987P, RRAR to GSAS (residues 682–685) at the furin cleavage site, a C-terminal T4 fibritin trimerization motif, an HRV3C protease cleavage site, a TwinStrep-tag and a 8x Hi- tag was synthesized and expressed using a CMV promoter. Sequence verified plasmids prepared using NucleoBond® Xtra Midi kit (Takara Bio USA, Inc) were transfected into 293 cells using FectoPro (Polyplus, USA). Proteins were purified from media 3–5 days post transfection using Protein A GraviTrap column or His GraviTrap column (GE Healthcare).

### ELISA affinity studies

The affinity of S-RBD or S1 for hACE2-Fc WT or variants was measured using a standard ELISA assay. Briefly, purified CoV2-S-RBD (2 μg/mL) or S1 (2 μg/mL) or the prefusion S-protein trimer (2 μg/mL) was coated onto 96-well ELISA plates (Fisher Scientific, #07-000-102) and incubated at 4 °C for 18 h. The coated plates were washed three times with 200 μL of PBST and then blocked with 200 μL of 3% BSA (Sigma-Aldrich # A8327) in PBST (Sigma-Millipore # 524653) and incubated for 1 h at room temperature. After washing the plates three times with 200 μL of PBST an increasing concentration of hACE2-Fc proteins were added and incubated for 1 h at room temperature. The unbound hACE2-Fc was removed by washing the plate three times with 200 μL of PBST. The bound hACE2 was detected using Goat-anti-human-IgG-Fc-HRP (Jackson Immuno Research # 109-035-008; 1:5000 dilution) using 50 μL TMB substrate (Pierce/ThermoFisher Scientific # 34028). After 3 min, the reaction was stopped using 50 µL of 2 N H_2_SO_4_. The optical density of the reaction was measured at 450 nm using a plate reader (Molecular Devices Gemini XPS). The data was analyzed and EC50 was calculate using Prism (GraphPad).

### Biolayer interferometry

To determine the binding kinetics and estimate the affinity (*K*_d_) of ACE2 variants for SARS-CoV-2 S-RBD, biolayer interferometry (BLI) experiments were performed on an Octet HTX instrument (ForteBio, USA) at 1000 rpm and 25 °C^72^. Biotinylated S-RBD protein was first captured on streptavidin biosensors from a 2 μg/mL solution to achieve binding response of 0.4–0.6 nm, followed by a quench step of 180 s with 100 μg/mL biotin. ACE2 variant proteins were diluted with assay buffer (PBS, 1% BSA, 0.05% Tween 20) and 200 nM of an unrelated biotinylated protein of similar size was used as negative control. After equilibrating with assay buffer, the loaded biosensors were dipped for 600 s into wells containing 3-fold serial dilutions of each variant from 200 nM and subsequently were transferred for 600 s back into assay buffer. Binding response data were reference subtracted and were fitted with 1:1 binding model using ForteBio’s Data Analysis software 9.0.

### Generation of SARS-CoV-2 pseudovirus

To generate SARS-Cov-2 pseudovirus, Human embryonic kidney 293 (HEK 293, ATCC^®^ CRL-1573™) cells were seeded at 0.3 × 10^6^ cells/well, in DMEM (ThermoFisher Scientific, Cat. No. 11995073) supplemented with 10% FBS and 1% Penicillin-Streptomycin (Gibco, Cat. No. 15070063) and grown overnight at 37 °C, 5% CO_2_. HEK 293 cells were then co-transfected with 1 μg of pNL4-3.luc.R-E- plasmid (luciferase expressing HIV-1 with defective envelop protein) (NIH AIDS Reagent Program, Cat. No. 3418) and 0.06 μg of CMV promoter-driven plasmid encoding SARS-CoV-2 wild-type or mutant spike variants using Lipofectamin™ 2000 transfection reagent (ThermoFisher Scientific, Cat. No. 11668027)^72^. Pseudoviruses harvested from the supernatant at 48 h post-transfection were filter sterilized (0.44 μm, Millipore Sigma, Cat. No. SLHA033SS) and used.

### Pseudovirus entry assay

HEK 293 cells (ATCC^®^ CRL-1573) stably over-expressing full-length human ACE2 protein were seeded in 96-well white polystyrene microplates (Corning, Cat. No. CLS3610) at 0.03 × 10^6^ cells/well in DMEM (10% FBS and 1% Pen-Strep), and grown overnight at 37 °C, 5% CO_2_. To test the inhibition of pseudovirus entry by ACE2 WT or mutants, increasing concentration of Fc-tagged ACE2 proteins were first mixed with pseudoviruses and incubated at room temperature for 10 m^72^. The ACE2 WT or mutant treated pseudovirus mixture was then used to infect cells. The cells were incubated at 37 °C, 5% CO_2_ for 6 h, then the medium was replaced with fresh DMEM (10% FBS and 1% Pen-Strep), and then again every 24 h for up to 72 h. To measure the luciferase signal (a proxy for virus uptake), DMEM was removed and cells were replaced in DPBS (ThermoFisher, Cat. No. 14190250) and mixed with an equal volume of ONE-Glo™ EX Luciferase Assay System (Promega, Cat. No. 8130). Relative luciferase units were measured using a BioTek Synergy Neo plate reader (BioTek Instruments Inc.). The data was then analyzed using GraphPad Prism Version 8.4.3 (GraphPad Software, LLC.).

### Statistics and reproducibility

Data were presented as the mean ± SD (standard deviation) of all experiments with *N* = number of biological replicates. Data were evaluated with the Wilcoxon test and Fisher’s exact test for statistical significance. Means were judged as statistically insignificant when *p* > 0.05. Box plots represent the median (center line), the interquartile range (IQR; box limits) and 1.5× IQR for the whiskers.

### Reporting summary

Further information on research design is available in the Nature Research Reporting Summary linked to this article.

## Supplementary information

Supplementary Information

Description of Additional Supplementary Files

Supplementary Data 1

Supplementary Data 2

Supplementary Data 3

Supplementary Data 4

Supplementary Data 5

Supplementary Data 6

Supplementary Data 7

Reporting Summary

## Data Availability

All ACE2 variant data used in this study were obtained from publicly available data sources as described in the methods. The ACE2 variant data is also presented in Supplementary Data 1.
