# Supplementary material for: Human ACE2 receptor polymorphisms and altered susceptibility to SARS-CoV-2
Source: Commun Biol. 2021 Apr 12;4:475. doi: 10.1038/s42003-021-02030-3 (PMC8041869; doi:10.1038/s42003-021-02030-3)
Supplement: Supplementary file 1 — Supplementary Information [file 42003_2021_2030_MOESM1_ESM.pdf]

Supplementary Information

Human ACE2 receptor polymorphisms and altered susceptibility to SARS-CoV-2

Kushal Suryamohan[1*], Devan Diwanji[2,3*], Eric W. Stawiski[1*], Ravi Gupta[4], Shane Miersch[5], Jiang Liu[6], Chao Chen[5], Ying-Ping Jiang[6], Frederic A. Fellouse[7], J. Fah Sathirapongsasuti[1], Patrick K. Albers[8], Tanneeru Deepak[4], Reza Saberianfar[7], Aakrosh Ratan[9, 10], Gavin Washburn[1], Monika Mis[1], Devi Santhosh[6], Sneha Somasekar[11], Hiranjith G. H.[1], Derek Vargas[1], Sangeetha Mohan[12], Sameer Phalke[12], Boney Kuriakose[13], Aju Antony[12], Mart Ustav[5], Stephan C. Schuster[10,14], Sachdev Sidhu[5], Jagath R. Junutula[6], Natalia Jura[2,3#], Somasekar Seshagiri[6,15#]

[1]Research and Development Department, MedGenome Inc., Foster City, CA 94404, USA; [2]Cardiovascular Research Institute, University of California San Francisco, San Francisco, CA 94158, USA; [3]Department of Cellular and Molecular Pharmacology University of California San Francisco, San Francisco, CA 94158, USA; [4]MedGenome Labs Ltd., 3rd Floor, Narayana Nethralaya Building, Narayana Health City, #258/A, Bommasandra, Hosur Road, Bangalore, Karnataka 560099, India; [5]Department of Molecular Genetics, and the Terrence Donnelly Center for Cellular and Biomolecular Research, University of Toronto, Toronto, Ontario M5S 3E1, Canada; [6]ModMab Therapeutics, 348 Hatch Drive, Foster City, CA 94404, USA; [7]ModMab Therapeutics, Accelerator for Donnelly Collaboration, University of Toronto, Toronto, Ontario M5S 1A8, Canada; [8]Wellcome Sanger Institute, Oxford, Hinxton, Cambridge, CB10 1SA UK; [9]Center for Public Health Genomics, University of Virginia, Charlottesville, VA, USA; [10]GenomeAsia100K Consortium; [11]Midwestern University, Glendale, AZ 85308; [12]Department of Molecular Biology, SciGenom Labs Pvt Ltd, Kerala 682037, India; [13]AgriGenome Labs Private Ltd, Kochi, Kerala 682030, India; [14]Singapore Centre for Environmental Life Sciences Engineering, Nanyang Technological University, Singapore, Singapore; [15]SciGenom Research Foundation, 3rd Floor, Narayana Nethralaya Building, Narayana Health City, #258/A, Bommasandra, Hosur Road, Bangalore, Karnataka, 560099, India.

* co-first author
# correspondence: NJ - natalia.Jura@ucsf.edu and SS – sekar@sgrf.org

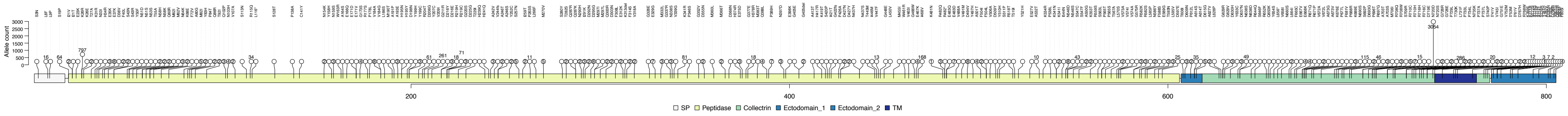

Supplementary Figure 1. Lollipop plot of ACE2 protein showing protein altering polymorphic variants observed across the entire protein. Allele counts for each polymorphism is shown inside or above each circle. Empty circles indicate singletons.

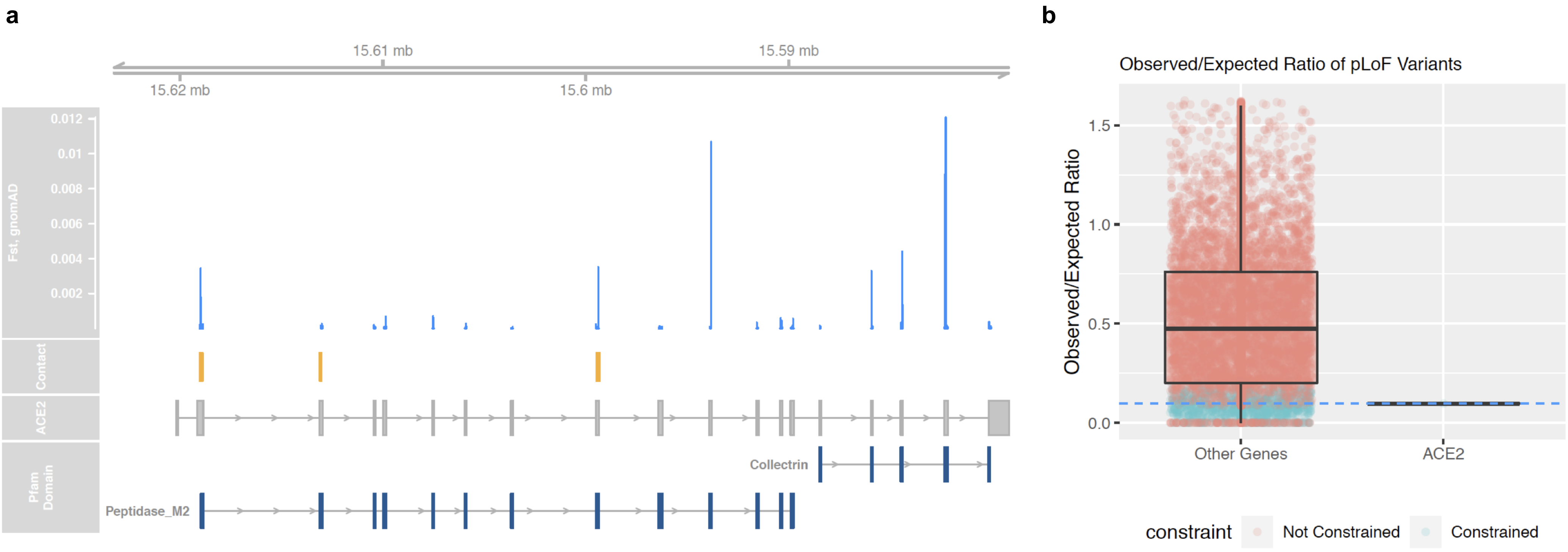

Supplementary Figure 2. (a) Fst index of exonic variants of ACE2, calculated from 57,783 female individuals across eight populations in gnomAD. Canonical transcript of ACE2 (ENST00000427411) and two Pfam domains are shown along with the positions of known SARS-CoV-2 contact residues. Peptidase domain harbor variants with lower variation (Wilcoxon p-value=0.0656; n=65). (**b)** ACE2 is highly constrained (pLI=0.9977), with the observed-to-expected ratio of the number of pLoF variants of 0.0968, consistent with the constrained genes (highlighted in cyan).

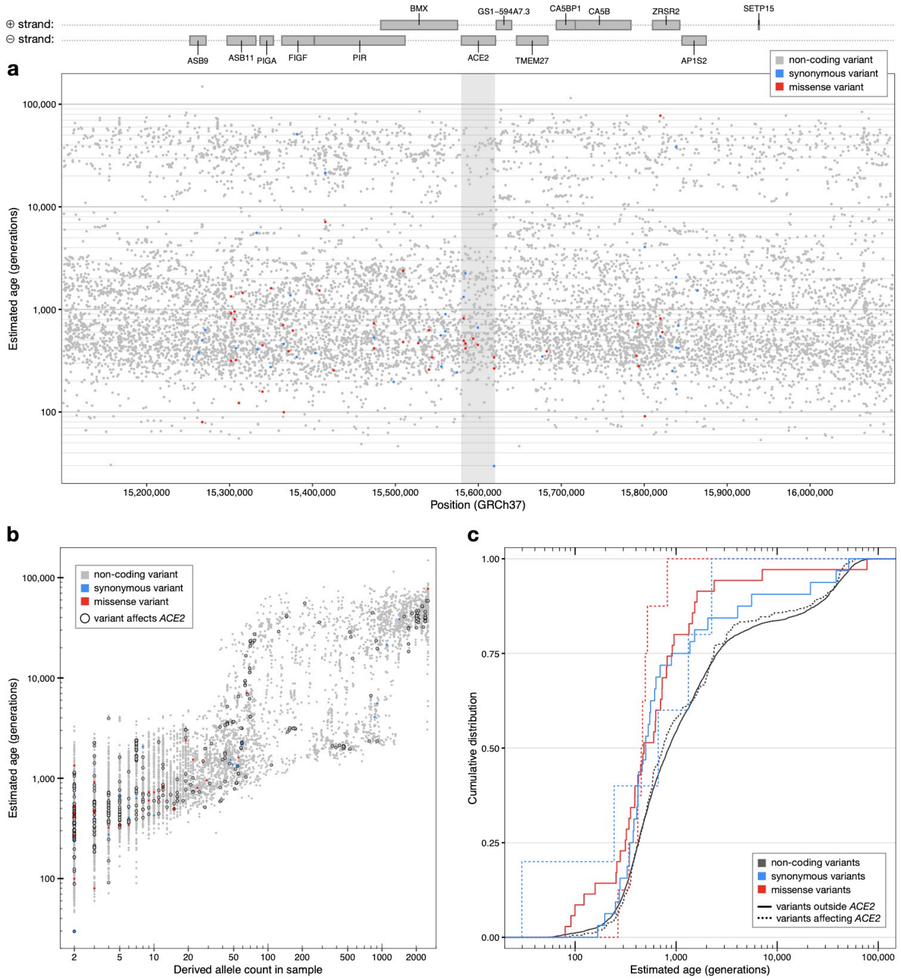

Supplementary Figure 3. Genealogical estimation of variant age (GEVA) analysis of variants in a 1 Mb region around the ACE2 gene; colors distinguish non-coding (gray), synonymous (blue), and missense (red) variants, predicted using the Ensembl Variant Effect Predictor (VEP) analysis. **(a)** Physical location (position on Chromosome X) and estimated age of the variants dated using GEVA; gene tracts (top) indicate the location of the larger genes within the region, highlighting the *ACE2* gene (shaded area). **(b)** Comparison between allele frequency (count of the derived allele in the sample) and estimated age; highlighting variants within (or VEP predicted effects on) the *ACE2* gene (black circles). **(c)** Empirical cumulative distribution of variants by estimated age, comparing variants outside the *ACE2* gene region (solid lines) to variants affecting *ACE2* (dashed lines).

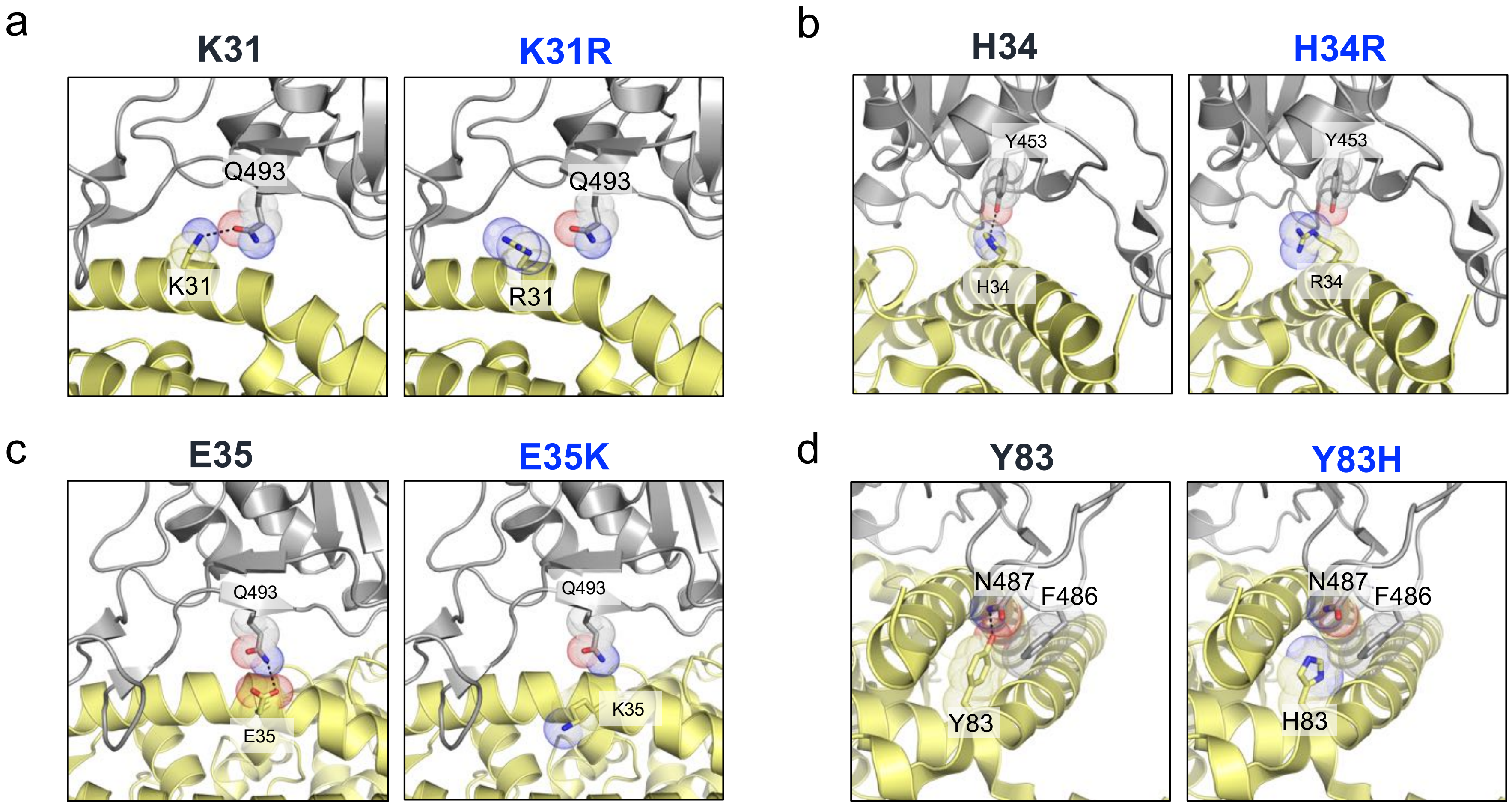

Supplementary Figure 4. Structural basis for the destabilizing effects of human ACE2 polymorphisms K31R, E35K, H34R, and Y83H on the interactions with SARS-CoV-2. (a) ACE2 K31R shows breaks an energetically favorable electrostatic interaction with Q493 of SARS-CoV-2 RBD. (b) ACE2 E35K breaks energetically favorable electrostatic interaction with Q493 of SARS-CoV-2 RBD. (c) ACE2 H34R removes electrostatic interaction with Y453 of SARS-CoV-2 RBD. (d) ACE2 Y83H removes polar contact with N487 as well as reduces hydrophobic packing with the unique SARS-CoV-2 RBD F486. All figures drawn from PDB ID: 6LZG.

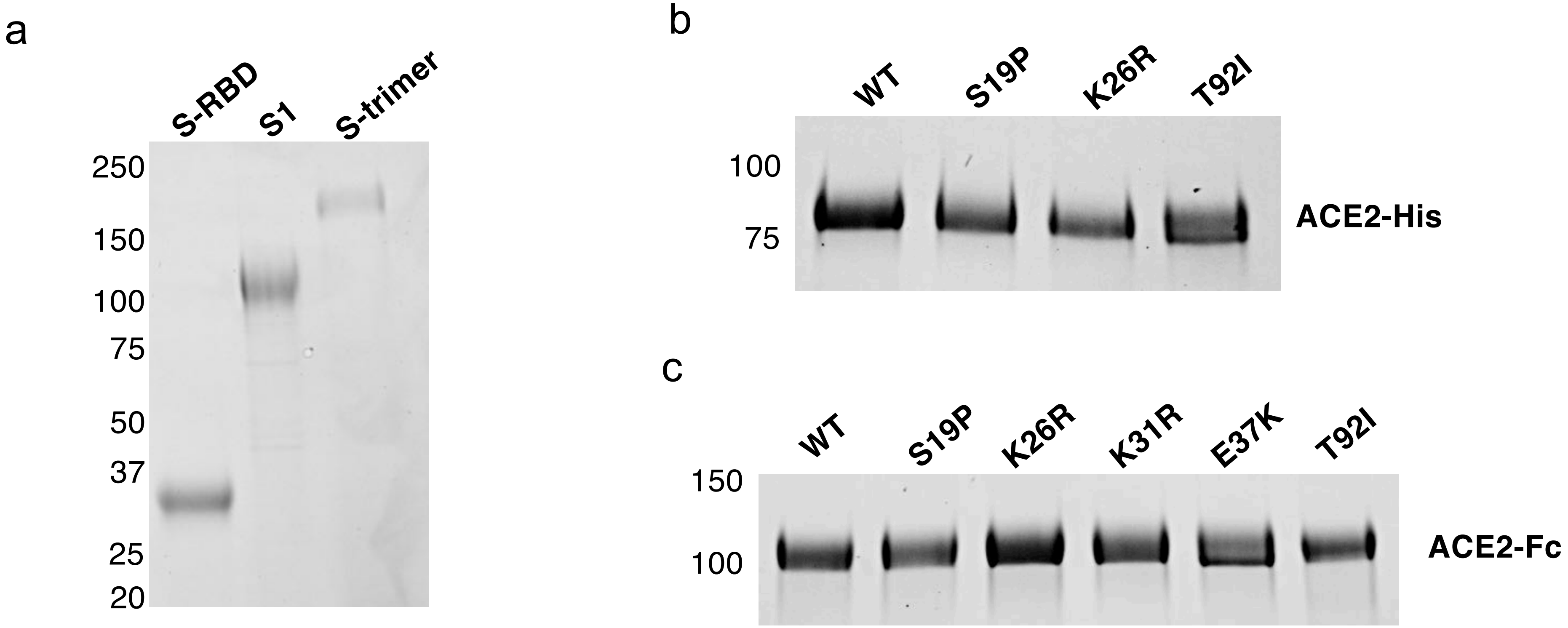

Supplementary Figure 5. (a-c) Purified recombinant S-protein and ACE2 were resolved on 4-15% SDS-PAGE (Mini-PROTEAN TGX Stain-Free Precast Gel).

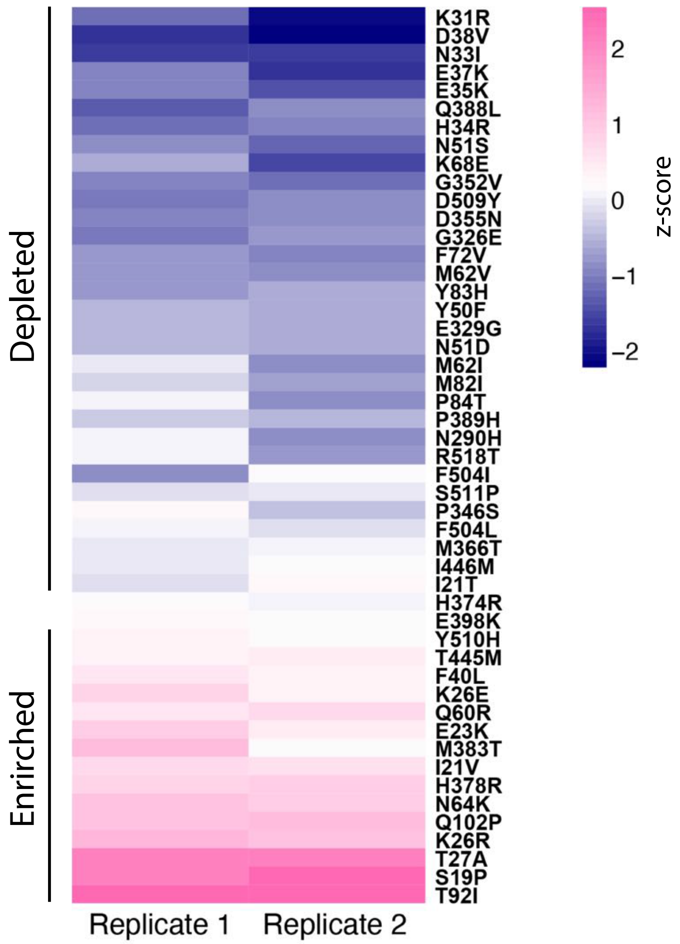

Supplementary Figure 6. Heatmap showing human ACE2 polymorphism that map to the ACE2-RBD interaction region and the corresponding enrichment/depletion scores from a recent study (*Science 2020, 10.1126/science.abc0870*).

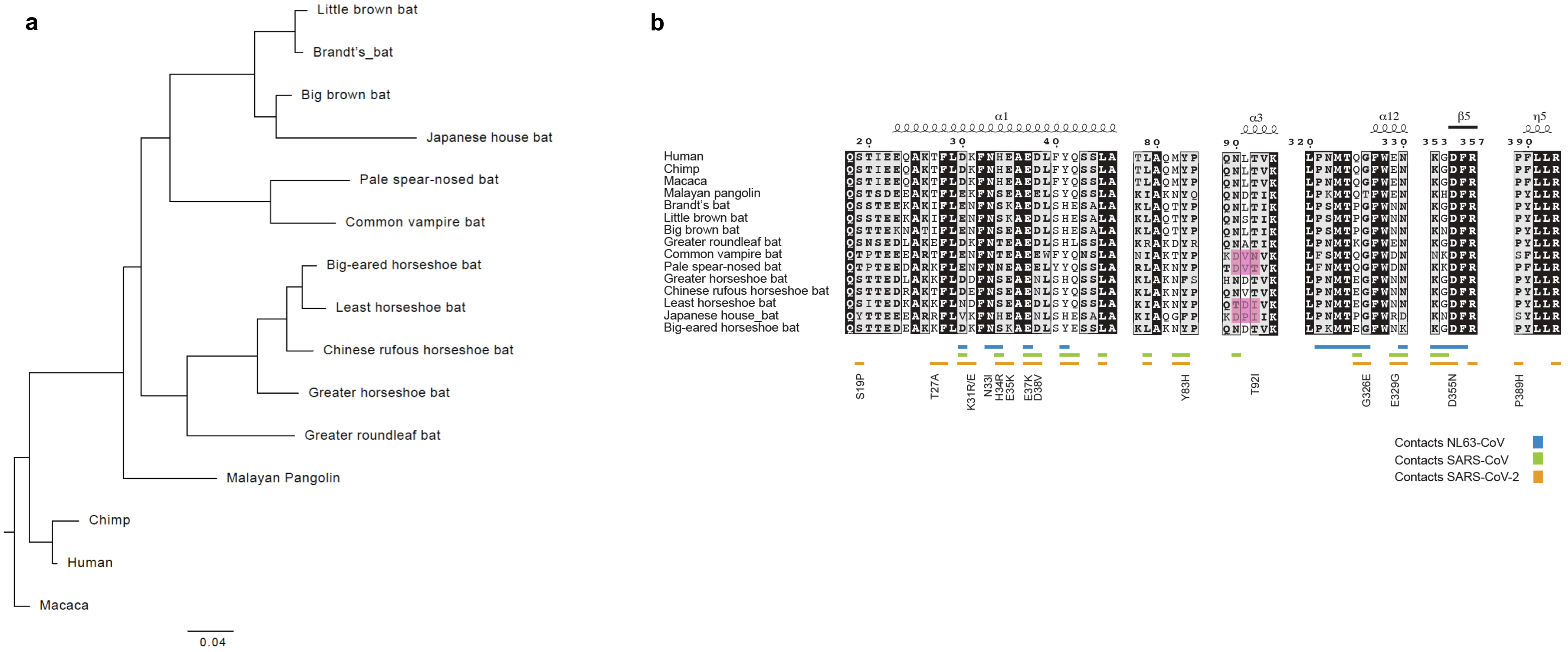

Supplementary Figure 7. **ACE2 sequence comparison** (**a**). Phylogenetic tree of ACE2 sequences from selected species, **(b)** Multiple sequence alignment of representative primate ACE2 sequences and ACE2 sequences of putative natural and intermediate reservoirs of coronaviruses. Pink boxes highlight species where the canonical NxT/S motif is absent or altered.
