# Supplementary material for: Human ACE2 receptor polymorphisms and altered susceptibility to SARS-CoV-2
Source: Commun Biol. 2021 Apr 12;4:475. doi: 10.1038/s42003-021-02030-3 (PMC8041869; doi:10.1038/s42003-021-02030-3)
Supplement: Supplementary file 2 — Description of Additional Supplementary Files [file 42003_2021_2030_MOESM2_ESM.pdf]

Description of Additional Supplementary Files

File name: Supplementary Data 1
Description: Human ACE2 protein altering population variants.

File name: Supplementary Data 2
Description: ACE2 polymorphisms predicted to alter SARS-CoV/Cov-2 S-protein binding.

File name: Supplementary Data 3
Description: Structure evaluation findings.

File name: Supplementary Data 4
Description: Conservation of N90 glycosylation motif in annotated jawed vertebrate ACE2 orthologs.

File name: Supplementary Data 5
Description: ELISA-based measurement of binding affinity of SARS-CoV-2 S-RBD, S1 and S-trimer.

File name: Supplementary Data 6
Description: Biolayer interferometry (BLI)-based determination of binding kinetics and affinity (Kd) of ACE2 variants for SARS-CoV-2 S-RBD

File name: Supplementary Data 7
Description: Pseudovirus entry inhibition assay
